# Aphonia induced by simultaneous bilateral ischemic infarctions of the putamen nuclei: a case report and review of the literature

**DOI:** 10.1186/1752-1947-7-83

**Published:** 2013-03-19

**Authors:** Vladimir V Senatorov, Shirish Satpute, Katherine Perry, David M Kaylie, John W Cole

**Affiliations:** 1 Department of Neurology, Baltimore Veterans Affairs Medical Center and University of Maryland School of Medicine, Baltimore, MD 21201, USA; 2 Department of Otolaryngology, Baltimore Veterans Affairs Medical Center and University of Maryland School of Medicine, Baltimore, MD, 21201, USA; 3 Department of Surgery, Division of Otolaryngology, Duke University School of Medicine, Durham, NC, 27710, USA

## Abstract

**Introduction:**

Isolated aphonia induced by acute stroke is a rare phenomenon with only a few cases reported in the literature.

**Case presentation:**

We report an unusual case of a 44-year-old African-American man with a history of hypertension, smoking and cocaine use who developed acute aphonia secondary to simultaneous ischemic infarctions of the bilateral putamen nuclei.

**Conclusion:**

We describe the clinical presentation of acute aphonia induced by bilateral putamen nuclei ischemic infarctions, correlating clinical symptoms with injury localization. We further highlight the anatomic and functional organization of the neural pathways involved.

## Introduction

Isolated aphonia induced secondary to simultaneous acute ischemic infarctions of the bilateral basal ganglia is a rare phenomenon. Most often, acute ischemic lesions involving the basal ganglia present with multiple symptoms including motor weakness and pseudobulbar signs, among others. We report an unusual case of a 44-year-old African-American man with a history of hypertension, smoking and cocaine abuse who presented with isolated acute onset aphonia after experiencing isolated ischemic infarctions involving the bilateral putamen nuclei.

## Case presentation

A 44-year-old African-American man with a previous medical history of uncontrolled hypertension (noncompliant with antihypertensive medications), smoking cigarettes, and chronic alcohol and cocaine abuse presented to an outside hospital with the complaint of altered speech that had waxed and waned for one week. On presentation to the outside hospital, his National Institutes of Health Stroke Scale (NIHSS) was 2 for isolated dysarthria. His blood pressure was 285/185mmHg. After treatment with hydralazine, his systolic blood pressure decreased to about 130mmHg and he became acutely aphonic. He remained alert and followed all commands, but was unable to produce speech. With maximal effort, our patient was able to produce short breathy sounds. His NIHSS increased to 9, primarily based upon his inability to verbally respond to questions. Given his severe speech deficit, our patient was treated with intravenous tissue plasminogen activator with a subsequent improvement in NIHSS, returning to 2. He was transferred to our hospital for further workup.

**Figure 1:**
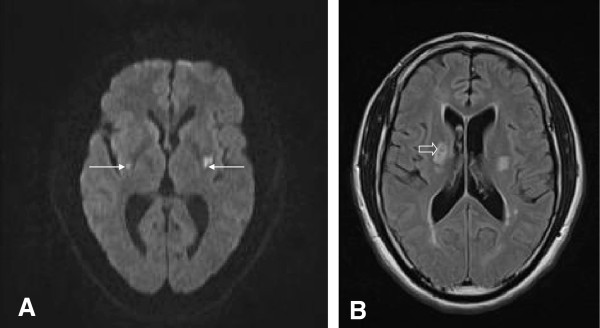
**Magnetic resonance imaging of the brain without contrast demonstrated areas of bilateral acute stroke within the lentiform nuclei. (A) **Diffusion-weighted image demonstrating acute stroke (arrows). **(B) **Fluid attenuated inversion recovery sequence demonstrating a more superior cut with evidence of prior stroke (open arrow) in the right basal ganglia.

On arrival at our hospital, our patient’s blood pressure was 169/98mmHg with a pulse of 86 beats per minute. Initial general physical and neurological examinations were unremarkable except for aphonia and mild right nasolabial flattening, with an NIHSS of 4 consistent with those deficits. The remainder of his neurological examination was non-focal. A toxicology screen was positive for urine cocaine metabolites. Our patient’s electrocardiogram demonstrated normal sinus rhythm with left atrial enlargement and ventricular hypertrophy, ST elevation, a prolonged QT interval and T-wave abnormality, consistent with possible inferior anterolateral ischemia. His level of troponin I was elevated. A transthoracic echocardiogram showed severe concentric left ventricular hypertrophy with impaired relaxation grade 1 diastolic dysfunction. Computed tomography of his brain revealed a chronic lacunar infarct in his right basal ganglia. Computed tomography angiography of his head and neck showed no evidence of vascular stenoses. Magnetic resonance imaging of his brain demonstrated bilateral areas of acute infarction within his lentiform nuclei on diffusion-weighted imaging (Figure 1A). The corresponding regions appeared dark on the apparent diffusion coefficient sequence (not shown). Specifically, both acute lesions were symmetrically located in the lateral part of the caudal aspect of his putamen. The right lesion was only visible in two axial magnetic resonance imaging slices, whereas the left lesion was visible in four axial slices. The maximal diameters of the diffusion-weighted imaging lesions in his right and left putamen nuclei were 5mm and 10mm, respectively.

These findings were compatible with acute ischemic infarcts within the regions supplied by the distal lateral lenticulostriate arteries. Magnetic resonance imaging fluid attenuated inversion recovery was also consistent with the acute ischemia seen on the diffusion-weighted imaging and apparent diffusion coefficient sequences, with a slightly more superior cut (Figure 1B) demonstrating the chronic lacunar infarct in his right basal ganglia, as seen on the initial head computed tomography. Our patient was hospitalized at our facility for five days; throughout this time he remained grossly aphonic and developed no further symptoms. He was started on daily statin and aspirin therapy and his blood pressure slowly normalized. He was counseled to quit smoking and discontinue all cocaine use. He was discharged home under 24-hour supervision.

While at home approximately two weeks after discharge, our patient experienced two episodes of transient right eye vision loss. The first episode lasted for approximately 30 minutes and resolved spontaneously; the second episode occurred two days later and our patient was again hospitalized. On presentation to our Emergency Department, he was hypotensive with a blood pressure of 101/68mmHg. His vision loss resolved spontaneously within five hours after aggressive hydration. Our patient's hypertensive medications, lisinopril and hydrochlorothiazide, were held. He was seen by our Ophthalmology Department, who suggested transient retinal ischemia in the setting of significant hypertensive retinopathy and potential medication-related hypotension. Our patient’s ability to speak had improved since his prior admission although his speech remained extremely hoarse and dysarthric. No other neurological deficits were noted. Our patient was evaluated by our Otolaryngology Department, who carried out an endoscopic examination and described decreased bilateral vocal fold (cord) function, more so on the right than left. Our patient was again discharged home with close follow-up.

At a follow-up visit to our neurology clinic approximately one month after the onset of the primary aphonic event, our patient continued to have moderately severe hoarseness and dysarthria, although his speech had improved in comparison to his second hospital admission. Our patient had experienced no further neurological events in the interim.

## Discussion

Acute unilateral infarctions limited to the lenticular nuclei are quite common and are often an accidental finding on imaging scans in patients with microangiopathy. Such lesions rarely cause clinical symptoms, representing just 1.6% of all deep cerebral infarcts leading to clinical symptoms of stroke [1]. Symmetrical acute lesions involving both basal ganglia as supplied by the perforating lenticulostriate arteries have rarely been reported. Such lesions have been associated with diffuse hypoxic injury [1], cardiogenic emboli [2] and cocaine or heroin toxicity [3]. The few reported cases that do describe acute bilateral infarctions as limited to both lenticular nuclei typically describe additional clinical deficits beyond aphonia, making our case unusual. To the best of our knowledge, there is only one prior report of a patient with isolated aphonia caused by simultaneous bilateral lenticulostriate artery territory infarctions [2].

**Figure 2:**
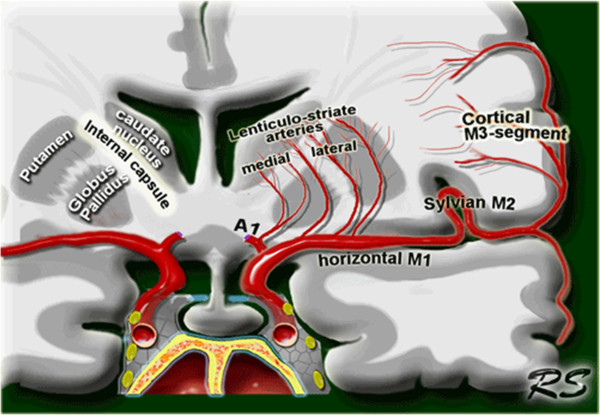
**Anatomical relationship between the lenticulostriate arteries and the lentiform nucleus.** This picture was reproduced from the Radiology Assistant website (http://www.radiologyassistant.nl) with permission from the author Dr Smithuis.

Our patient had several significant risk factors for stroke, including smoking, long-standing hypertension with medication noncompliance and cocaine use. These factors almost certainly contributed to the accumulation of diffuse pathological vessel changes, resulting in microangiopathy. During both instances in which our patient was hospitalized for acute symptomatology, he either developed or presented with hypotension. The sharp drop in blood pressure invoked by the treatment of hypertension in the Emergency Department likely induced an abrupt local hypoperfusion within the distal segments of his deep brain-penetrating lenticulostriate arteries (Figure 2). In the setting of hypotension, the brain tissue supplied by these penetrating ‘end vessels’ would have been the most vulnerable because there was no collateral blood supply. This made them more susceptible to hypoperfusion, further exacerbated by our patient’s preexisting microangiopathy. Additionally, our patient’s recent cocaine use may have worsened the local ischemia via disruption of cerebral autoregulation [4].

**Figure 3:**
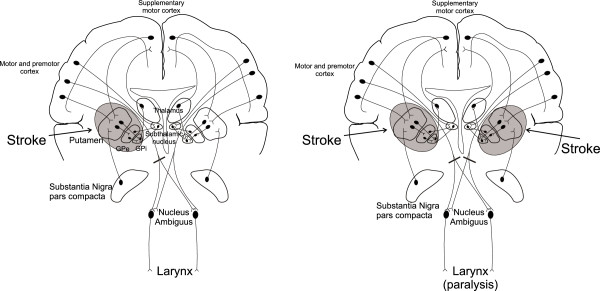
**Anatomical pathways between the cortex, basal ganglia, brainstem and vagus nerves innervating the vocal folds. **Pathway injury indicated by solid bars; **(A)** unilateral lesions do not present with vocal fold paralysis, while **(B) **bilateral lesions present with aphonia. GPe, globus pallidus external; GPi, globus pallidus interna.

Functionally, the production of sound (phonation) depends on intact function of the vocal folds and a complex interplay between the cerebral cortex, basal ganglia and brainstem nuclei (Figure 3). Feedback loops exist between the various subcomponents of the lentiform nuclei, primary motor cortex and thalamus. Specifically, the putamen receives projections from several cortical motor areas, including the supplementary motor area, ventral premotor cortex and sensorimotor cortex, and sends efferent projections to the internal segment of the globus pallidus. The ventrolateral nucleus of the thalamus receives input from the internal segment of the globus pallidus and closes the loop by feeding back to the cortex. Thus, isolated bilateral lesions of the putamen interrupt the flow of impulses in the striato-pallido-thalamo-cortical loop and to the phonatory motor nuclei (nucleus ambiguus), resulting in vocal fold paralysis. More commonly, the relationship between the basal ganglia and vocal cord innervations are evident in hypophonia - a symptom immediately recognized by clinicians evaluating patients with Parkinson’s disease or unilateral ischemic stroke.

Somatotopical organization of the putamen has been relatively well-studied in electrophysiological and neuroanatomical experiments involving the monkey [5]. To summarize, the motor territory of the striatum belongs to the caudal aspect of the putamen and only the lateral putamen receives projections from the primary motor cortex. In the lateral putamen, the corticostriatal projections from hindlimb, forelimb and orofacial representations are arranged from dorsal to ventral. The caudal putamen projects to the ventral two-thirds of the caudal globus pallidus. Neurons responding to the orofacial, forelimb and hindlimb regions of the primary motor cortex are located along the ventral-to-dorsal axis in the external and internal segments of the globus pallidus. The internal segment of the globus pallidus sends output to the oral part of the ventrolateral thalamic nucleus. The ventrolateral thalamic nucleus then sends fibers back to the primary motor cortex. Although much less is known about the somatotopy of the human putamen, imaging studies suggest similar somatotopical organization [6]. Available data correspond well with the locations of the lesions and the symptoms presented in our case.

Vocal fold paralysis induced by stroke is most commonly associated with ischemia in the brainstem; however, isolated vocal paralysis is an uncommon manifestation of stroke [7]. The vocal folds are regulated by the Xth cranial nerve (vagus), with the muscles of the larynx (voice box) controlled by the branchial motor component of the vagus originating in the nucleus ambiguus in the medulla. As there is no direct connection from the lentiform nuclei to the nucleus ambiguus, the upper motor neuron innervations to the nucleus ambiguus controlling voice are provided by the corticobulbar tract originating from the bilateral laryngeal motor cortex [8,9].

Because the nucleus ambiguus receives bilateral input from the motor cortex, a unilateral lesion of the corticobulbar tract prior to the ambiguus nucleus should not paralyze the unilateral vocal fold. Although unilateral corticobulbar tract lesions are not typically associated with vocal fold palsies, there are several cases reported describing altered phonation with injury to a dominant hemispheric projection from the contralateral corticobulbar tract to the vocal fold. For instance, Takase *et al*. [10] reported a right vocal fold paresis caused by hypoperfusion of the left hemisphere in the setting of severe stenosis of the left middle cerebral artery (MCA). Interestingly, in our patient, we found more decreased function in the right vocal fold as a possible consequence of a larger lesion in the left putamen.

As described, both acute lesions were symmetrically located in the posterolateral region of the putamen nuclei, near the posterior limb of the internal capsule. However, there was no indication that the lesions might have affected the internal capsule. There is some controversy regarding the somatotopy of the pyramidal tract in the human internal capsule, and experimental studies have shown the lack of a direct corticoambigual projection in rhesus monkey as well as in several other animal species [11]. Many believe that direct corticoambigual projections are a very recent acquisition in hominid evolution, reflecting speech development in humans. Current consensus is that corticospinal fibers of hand and foot representation pass in the posterior limb of the internal capsule; however, the pyramidal tract does not maintain a fixed position in the internal capsule and is shifting from the middle part of the posterior limb in the superior portion of the capsule to the posterior third of the posterior limb in the inferior portion of the capsule [12]. At the same time, the corticobulbar tract of head and neck representation occupies the genu [8,9]. The disruption of the corticobulbar tract by focal ischemic lesions in the genu of the internal capsule has been found to cause orofacial and laryngeal paresis [9]. Simonyan *et al*. [8], who studied spasmodic dysphonia (a neurological disorder characterized by involuntary spasm in the laryngeal muscles during speech production) using diffusion tensor imaging and neuropathological studies, reported bilateral changes in both the lentiform nucleus and the right genu of the internal capsule. The ischemic lesions in our case are close to the posterior portion of the posterior limb of the internal capsule, but far from the genu. If there was involvement of the internal capsule, it would produce symptoms associated with corticospinal injury but not corticobulbal tract lesions.

Thus, the isolated bilateral lentiform nuclei infarctions seen in our patient appear to have induced the vocal fold paralysis. As such, our case indicates that the putamen, and specifically its caudal part, appears to be necessary for phonation independent of any other injury. Physicians observing voice changes associated with weakness or paralysis of the vocal folds should consider possible injury to the lentiform nuclei of the brain. Lastly, physicians evaluating patients with stroke should be aware of the risks of overly aggressive treatment of hypertension in patients with acute stroke and a high risk for cerebral microangiopathy with multiple poorly controlled vascular risk factors.

## Conclusion

We describe the clinical presentation of acute aphonia induced by bilateral infarctions of the putamen, further correlating clinical symptoms with injury localization. We highlight the anatomic and functional organization of the neural pathways involved, suggesting that the regions of the lentiform nuclei supplied by the lateral lenticulostriate arteries may be necessary for vocal cord function, and that these regions appear to be particularly sensitive to fluctuations in blood pressure and the toxic effects of cocaine.

## Consent

Written informed consent was obtained from the patient for publication of this case report and accompanying images. A copy of the written consent is available for review by the Editor-in-Chief of this journal.

## Competing interests

The authors declare that they have no competing interests.

## Authors’ contributions

VVS, SS, KP and JWC all cared for the patient in both the inpatient and outpatient settings. DMK reviewed the case and provided clinical management suggestions. All authors contributed to the writing of the manuscript and read and approved the final version of the manuscript.

## References

[B1] Russmann H Vingerhoets F Ghika J Maeder P Bogousslavsky J Acute infarction limited to the lenticular nucleus. Clinical, etiologic, and topographic features Arch Neurol 2003 60 351 355 10.1001/archneur.60.3.351 12633146

[B2] Akkus DE Pure mutism due to simultaneous bilateral lenticulostriate artery territory infarction CNS Spectr 2006 11 257 259 16641830 10.1017/s1092852900020745

[B3] Daras MD Orrego JJ Akfirat GL Samkoff LM Koppel BS Bilateral symmetrical basal ganglia infarction after intravenous use of cocaine and heroin Clin Imaging 2001 25 12 14 10.1016/S0899-7071(00)00232-1 11435032

[B4] Treadwell SD Robinson TG Cocaine use and stroke Postgrad Med J 2007 83 389 394 10.1136/pgmj.2006.055970 17551070 PMC2600058

[B5] Nambu A Somatotopic organization of the primate basal ganglia Front Neuroanatomy 2011 5 1 8 10.3389/fnana.2011.00026 PMC3082737 21541304

[B6] Gerardin E Lehéricy S Pochon J-P Tézenas Du Montcel S Mangin JF Poupon F Agid Y Le Bihan D Marsault C Foot, hand, face and eye representation in the human striatum Cereb Cortex 2003 13 162 169 10.1093/cercor/13.2.162 12507947

[B7] Merati AL Heman-Ackah YD Abaza M Altman KW Sulica L Belamowicz S Common movement disorders affecting the larynx: a report from the neurolaryngology committee of the AAO-HNS Otolaryngol Head Neck Surg 2005 133 654 665 10.1016/j.otohns.2005.05.003 16274788

[B8] Simonyan K Tovar-Moll F Ostuni J Hallett M Kalasinsky VF Lewin-Smith MR Rushing EJ Vortmeyer AO Ludlow CL Focal white matter changes in spasmodic dysphonia: a combined diffusion tensor imaging and neuropathological study Brain 2008 131 447 459 10.1093/brain/awm303 18083751 PMC2376833

[B9] Bogousslavsky J Regli F Capsular genu syndrome Neurology 1990 40 1499 1502 10.1212/WNL.40.10.1499 2215938

[B10] Takase K Shigeto H Furuta K Sakae N Ohyagi Y Kira J Transient vocal cord palsy caused by hypoperfusion of unilateral hemisphere FukuokaIgaku Zasshi 2011 102 273 276 22111335

[B11] Simonyan K Jürgens U Efferent subcortical projections of the laryngeal motocortex in the rhesus monkey Brain Res 2003 974 43 59 10.1016/S0006-8993(03)02548-4 12742623

[B12] Kretschmann HJ Localisation of the corticospinal fibres in the internal capsule in man J Anat 1988 160 219 225 3253257 PMC1262065

